# Therapeutics

**Published:** 1880-08

**Authors:** 


*Collaborators:*

DR. L. W. CASE, DR. R. TILLEY.

### THERAPEUTICS.

THE SILK OF THE CORNSTALK IN THE TREATMENT OF AFFECTIONS OF THE BLADDER.—By Dr. Dassum.—(*L' Union Médicale*, April 5, 1880.)

Since the first article published by this journal (*L' Union Médicale*) numerous observations have been published, which clearly show the value of this article in some of the affections of the bladder. We extract the following from the published reports:

Report of Dr. Dezotteux.—Retention of urine by a man of 70 years. The catheter was used the first evening and the following: the sryup of the stigma of mais, given in table-spoonful doses every four hours, and the next morning the urine passed normally. The syrup was continued for some days, and cure was complete.

Ibid. Cystitis, dysuria. Urine ammonical in a man of 68 years. Three doses of the syrup produced a marked improvement, and eight days effected complete relief.

Report of Dr. Lamy. Retention of urine dating ten years in a man of 78, who was accustomed to catheterise himself. One evening, after a full supper, he could not pass the sound, and finding himself bleeding considerably, Dr. Lamy was called, who succeeded, after great difficulty, in withdrawing the urine. For fifteen days the urine was withdrawn by means of the catheter. It had a strong ammoniacal odor, and exhibited morbid deposits. The bladder was washed with carbolized solutions every day. Alkaline drinks were administered and inunctions of belladonna and gelsemium practiced, but all without result. The infusion* of the cornstalk was then administered. From the second day the urine was passed normally and the infusion having been continued for a few days, the retention of ten years disappeared.

Ibid. Retention of urine for twenty years, in a man of 83 who had used the catheter daily. In using the catheter, violent tenesmus occurred and blood followed. Dr. L administered the infusion of stigma of mais, and after the third dose the tenesmus ceased. During the night the urine was passed, quite colored with blood. The next day the blood had disappeared, and permanent relief followed.

Report of Dr. André. A man, 42 years, has suffered ten years from chronic cystitis, a sequel of gleet. During this time he has complained of pains in the lower part of the abdomen, a burning sensation when passing water, and a bearing down sensation in the rectum and perineum. The urine deposited mucus and uric acid. During the last three years there has existed a constant and imperative desire to urinate. The urine soon became ammoniacal, and finally it passed involuntarily. The classic treatment employed during the ten years was without result. The syrup in question was administered; no other medication. Eight days after, he went to the doctor's office completely relieved.

Dr. Dassum adds that the best mode of administering it, is in the form of syrup made from the extract; three tablespoonsful during the day in water. The syrup represents 27 grams of the extract to the liter.

The value of the silk seems to differ according to the way in which it has been collected or dried.

NOTE.—The French journals have published a good deal of late about the value of the corn silk, but no definite investigation seems to have been made of the active principle.

---

* Fifteen grammes in 500 c. c. of water during the day.

